# CO_2_ Hydrogenation over Nanoceria-Supported Transition Metal Catalysts: Role of Ceria Morphology (Nanorods versus Nanocubes) and Active Phase Nature (Co versus Cu)

**DOI:** 10.3390/nano9121739

**Published:** 2019-12-06

**Authors:** Michalis Konsolakis, Maria Lykaki, Sofia Stefa, Sόnia A. C. Carabineiro, Georgios Varvoutis, Eleni Papista, Georgios E. Marnellos

**Affiliations:** 1 School of Production Engineering and Management, Technical University of Crete, GR-73100 Chania, Greece; mlykaki@isc.tuc.gr (M.L.); sstefa@isc.tuc.gr (S.S.); 2 Laboratório de Catálise e Materiais (LCM), Laboratório Associado LSRE-LCM, Faculdade de Engenharia, Universidade do Porto, 4200-465 Porto, Portugal; 3 Department of Mechanical Engineering, University of Western Macedonia, GR-50100 Kozani, Greece; georgios.varvoutis@gmail.com (G.V.); lpapista@gmail.com (E.P.); gmarnellos@uowm.gr (G.E.M.); 4 Chemical Process & Energy Resources Institute, Centre for Research & Technology Hellas, GR-57001 Thermi, Thessaloniki, Greece

**Keywords:** CO_2_ hydrogenation, copper, cobalt, nanoceria, reverse water-gas shift reaction, methanation (Sabatier) reaction

## Abstract

In this work we report on the combined impact of active phase nature (M: Co or Cu) and ceria nanoparticles support morphology (nanorods (NR) or nanocubes (NC)) on the physicochemical characteristics and CO_2_ hydrogenation performance of M/CeO_2_ composites at atmospheric pressure. It was found that CO_2_ conversion followed the order: Co/CeO_2_ > Cu/CeO_2_ > CeO_2_, independently of the support morphology. Co/CeO_2_ catalysts demonstrated the highest CO_2_ conversion (92% at 450 °C), accompanied by 93% CH_4_ selectivity. On the other hand, Cu/CeO_2_ samples were very selective for CO production, exhibiting 52% CO_2_ conversion and 95% CO selectivity at 380 °C. The results obtained in a wide range of H_2_:CO_2_ ratios (1–9) and temperatures (200–500 °C) are reaching in both cases the corresponding thermodynamic equilibrium conversions, revealing the superiority of Co- and Cu-based samples in methanation and reverse water-gas shift (rWGS) reactions, respectively. Moreover, samples supported on ceria nanocubes exhibited higher specific activity (µmol CO_2_·m^−2^·s^−1^) compared to samples of rod-like shape, disclosing the significant role of support morphology, besides that of metal nature (Co or Cu). Results are interpreted on the basis of different textural and redox properties of as-prepared samples in conjunction to the different impact of metal entity (Co or Cu) on CO_2_ hydrogenation process.

## 1. Introduction

It is widely accepted amongst the scientific community that the increasing trend of CO_2_ emissions in the Earth’s atmosphere since the onset of industrialization is the key attributor for the planet temperature rise over the last two centuries [1]. Global temperature is projected to rise by the year 2040 by 1.5 °C in comparison with the pre-industrial levels, according to the latest Intergovernmental Panel on Climate Change (IPCC) report on the impacts on global warming [2,3]. Efforts of mitigation of the aforementioned environmental issue can be simplified into three general approaches: (i) complete and/or partial replacement of carbon-based fuels with renewable energy sources (RESs), (ii) carbon dioxide capture and storage (CCS) technology and (iii) chemical conversion/utilization of CO_2_ toward value-added chemicals and fuels [4].

The latter approach has attracted intense interest over the past decades, with hydrogenation of CO_2_ being one of the most thoroughly investigated methods, owing to the wide variety of possible products [5]. This route can also provide an effective way to valorize CO_2_ emissions and efficiently store the surplus power from non-intermittent RESs (e.g., solar, wind) in the form of “green” hydrogen, providing either CO via the mildly endothermic reverse water-gas shift (rWGS) reaction (Equation (1)) or CH_4_ via the highly exothermic methanation reaction, often referred to as the “Sabatier reaction” (Equation (2)), discovered in 1902 by the French scientist Paul Sabatier [6].

CO_2_ + H_2_ ↔ CO + H_2_O, ΔH_298K_ = +41.3 kJ/mol (1)

CO_2_ + 4H_2_ ↔ CH_4_ + 2H_2_O, ΔH_298K_ = −164.7 kJ/mol (2)

Other possible products of potential value from CO_2_ hydrogenation include methanol [7,8,9,10], dimethyl ether [11], formic acid [12] and hydrocarbons [13].

Among the different CO_2_ hydrogenation products, carbon monoxide is a valuable feedstock for the C1 chemical industry, since various liquid synthetic hydrocarbons and chemicals can be produced by its subsequent upgrading, through the well-established Fischer–Tropsch synthesis [14,15]. Moreover, CO can be used in nickel purification [16]. Methane, on the other hand, as a major constituent of natural gas, is an energy carrier whose significance is applicable globally from household use to the industrial, energy, and transportation sectors. Given that the hydrogen needed for carbon dioxide methanation is provided via a carbon-neutral energy source (e.g., solar-powered water electrolysis), the overall scheme can be labeled as a power-to-gas (PtG) process [17,18]. Carbon dioxide methanation can effectively convert a less manageable energy vector, such as gaseous hydrogen into a high-energy source such as methane, since CH_4_ possesses three times the volumetric energy density of hydrogen [19]. Also, CH_4_ can be easily integrated into the existing natural gas storage and distribution network, especially throughout Europe [20,21].

Regarding the overall sustainability of the proposed process, it should be pointed out that the general scheme of the CO_2_ hydrogenation concept is associated with the use of “green” H_2_, originated by the excess energy provided by RESs and the concentrated amounts of CO_2_ emissions as feedstock. In this regard, the CO_2_ hydrogenation process could be implemented near a source of highly concentrated CO_2_ emissions, such as effluent streams of the steel industry or a CO_2_ capture plant. By employing highly active and inexpensive catalysts, large amounts of CO_2_ can be potentially mitigated with the concurrent production of value-added products, such as CH_4_ or CO, which can be used as fuels or feedstock in the chemical industry. Regarding the use of renewable hydrogen, it can be exploited entirely in the hydrogenation process or it can be partially converted to the electricity required for the reaction process. Also, excess hydrogen can be directly injected into the gas grid or used in fuel cell-powered vehicles. Various comprehensive studies have been devoted to the sustainability of the CO_2_ hydrogenation process, to which the reader can refer [22,23,24,25,26].

Whereas hydrogenation of CO_2_ can be a promising way to reduce the environmental carbon print, several limitations arise for the implementation of a technology based on either Equation (1) or Equation (2). The first is associated with the activation of carbon dioxide itself, a fully oxidized and thermodynamically stable compound whose reduction is not energetically favorable [27], and thus requires strong reductants (i.e., H_2_) or electrochemical-assisted reduction processes [28,29]. Secondly, CO_2_ hydrogenation is subjected to kinetic and equilibrium limitations, thus reaction rates need to be promoted [30]. Numerous catalytic systems have been employed in order to overcome these limitations and several reviews in the literature summarize the catalysts explored for either rWGS [2,31,32] or CO_2_ methanation [33,34,35,36] reactions. The most studied catalytic systems are composites with metals supported on reducible metal oxides (e.g., CeO_2_, ZrO_2_) or a combination of them. These systems have been employed as bi-functional catalysts, with oxide supports mainly providing oxygen vacancies to activate CO_2_, and metal active sites dissociating molecular hydrogen, the so-called hydrogen spillover process [31,37].

Among the oxides investigated, CeO_2_ has attracted much attention as a supporting carrier, due to its high oxygen mobility and unique redox properties, as cerium can rapidly change between its two oxidation states (Ce^3+^ and Ce^4+^) [38,39]. Moreover, ceria is a basic oxide promoting a strong interaction with CO_2_, facilitating its adsorption [40]. Besides bare ceria’s excellent redox properties, many studies have focused on the development of highly efficient and low-cost ceria-based catalytic composites, since the combination of various non-noble transition metals (TMs) (e.g., Cu, Co, Ni, Fe) with ceria, can further enhance the catalytic activity and/or selectivity due to the peculiar metal-support synergistic interactions [41,42,43]. Despite their adequate catalytic activity, the use of precious metals like Ru [44], Rh [45] and Pd [46] as active phases is generally not preferable, since their high cost and scarcity might render the process financially non-viable. Thus, from a techno-economical point of view, the use of TMs-based catalysts may be favorable, since these metals can achieve comparable activity to that of the most active noble metal catalysts albeit at a substantially lower cost [20,47]. In this regard, efforts from our group have recently focused on developing inexpensive TMs-based catalytic composites, with particular emphasis on middle-late 3d metals, i.e., Cu, Co, Ni, Fe, which were found to adsorb and consequently activate CO_2_ through a charge transfer from metal phase to CO_2_ moiety [48]. In particular, Density Functional Theory (DFT) calculations have revealed spontaneous chemisorption of CO_2_ and favorable thermodynamic properties for the aforementioned metals, with Cu, however, exhibiting a weaker interaction with CO_2_ [48].

In order to develop highly efficient ceria-based catalysts, much research has been devoted on the rational design of catalytic materials by means of advanced synthetic and/or promotional routes [49,50,51,52]. The ultimate goal would be to obtain catalytic systems with adequate stability, CO_2_ conversion activity and high product selectivity, in order to exclusively generate CO or CH_4_ in a real large-scale process. Of major importance towards fine-tuning of CeO_2_-based materials is the decrease of particles size in the nanoscale. Nano-materials exhibit abundance in surface atoms and defect sites such as oxygen vacancies, whereas the electronic perturbations between the metal and support nanoparticles greatly affect the catalytic performance [41,53,54]. Moreover, by tailoring the shape of nanoparticles by means of advanced synthetic routes (e.g., hydrothermal method), different crystal facets can be exposed leading to different oxygen storage capacity (OSC) and oxygen mobility [55,56]. For example, Ouyang et al. [57] have investigated ceria morphological effects during methanol synthesis from CO_2_ hydrogenation over CuO/CeO_2_ mixed oxides; copper-ceria nanorods exhibited the highest CO_2_ conversion and methanol yield due to the strongest metal–support interaction, as compared to nanocubes and nanospheres. Similarly, Au/CeO_2_ nanorods were found to exhibit a stronger gold-ceria interaction and higher activity in the forward WGS reaction than cubic and polyhedral CeO_2_ [58]. Also, Liu et al. [59] reported a better activity for ceria nanocubes in the rWGS reaction than nanorods and nanopolyhedra, with the nanocubic samples prepared, preferentially exposing (100) planes, a potentially more active surface than (110) and (111) planes.

Although there are several studies regarding the hydrogenation of CO_2_ over ceria-based composites, it should be noted that CO_2_ hydrogenation proceeds through a complex reaction pathway, being affected to a different extent by various factors, such as the metal–oxide interactions, the formation of oxygen vacancies, the reducibility, etc. [51,60,61,62,63,64]. Furthermore, selectivity towards CO, CH_4_ or other possible compounds can vary remarkably when using catalytic composites with various active metal phases supported onto ceria, depending thus on the metal entity employed [65,66,67].

In light of the above, the aim of the present study is to investigate the effect of non-noble metal phase nature (Co, Cu) and support morphology (nanorods, nanocubes) on the textural, structural, redox properties and, consequently, on the CO_2_ hydrogenation performance of mesoporous ceria-based nanocatalysts. The originality of the present work relies on the combined impact of transition metal nature (Cu, Co) and support morphology (ceria nanorods or nanocubes) on the CO_2_ hydrogenation performance over a wide range of H_2_:CO_2_ ratios (1–9) and temperatures (200–500 °C), in conjunction with the thermodynamic analysis performed under different reaction conditions. The as-prepared samples were synthesized hydrothermally and characterized by N_2_ adsorption-desorption, X-ray diffraction (XRD), transmission electron microscopy (TEM) and temperature programmed reduction (TPR) methods and they were catalytically evaluated in the CO_2_ hydrogenation reaction at atmospheric pressure. The obtained results are interpreted on the basis of a thermodynamic analysis at different reaction conditions, in conjunction to structural and surface characterization results that reveal the key role of metal entity and support morphology both on CO_2_ conversion and products selectivity.

## 2. Materials and Methods

### 2.1. Reagents

All chemicals used were of analytical reagent grade. Ce(NO_3_)_3_·6H_2_O (purity ≥ 99.0%, Fluka, Bucharest, Romania), Cu(NO_3_)_2_·2.5H_2_O (Fluka, Bucharest, Romania) and Co(NO_3_)_2_·6H_2_O (≥98%, Sigma-Aldrich, St. Louis, MO, USA) were used as precursors for the preparation of bare ceria, Cu/CeO_2_ and Co/CeO_2_ catalysts, respectively. Sodium hydroxide (≥98%, Honeywell Fluka, Bucharest, Romania), ethanol (purity 99.8%, ACROS Organics, Waltham, MA, USA) and double deionized water (DI) were also employed during catalysts preparation procedure.

### 2.2. Materials Synthesis

Initially, bare ceria nanoparticles were synthesized by the hydrothermal method, as described in our previous work [68]. Briefly, for the synthesis of bare ceria nanostructures, appropriate amounts of Ce(NO_3_)_3_·6H_2_O and NaOH were initially dissolved in double deionized water, then mixed under vigorous stirring for 1 h and aged for 24 h at 90 °C for ceria nanorods and at 180 °C for ceria nanocubes. Eventually, the resulting solids were recovered by centrifugation, washed thoroughly with double deionized water until pH reached a value of 7, in order to remove any co-precipitated salts, and finally washed with ethanol to avoid agglomeration of the nanoparticles. Then, the precipitate was dried at 90 °C for 12 h, followed by calcination at 500 °C for 2 h, under air flow (heating ramp 5 °C/min). The bare ceria samples are designated as CeO_2_-NX (NX: NR (nanorods), NC (nanocubes)).

Cu/CeO_2_-NX and Co/CeO_2_-NX catalysts were synthesized by the wet impregnation method, using aqueous solutions of Cu(NO_3_)_2_·2.5H_2_O and Co(NO_3_)_2_·6H_2_O, respectively, in order to obtain a metal/cerium atomic ratio of 0.25, corresponding to a Cu loading of ca. 8.5 wt.% and a Co loading of ca. 7.9 wt.%. Then, the obtained suspensions were heated under stirring until water evaporation, dried at 90 °C for 12 h and finally calcined at 500 °C for 2 h under air flow (heating ramp 5 °C/min).

### 2.3. Materials Characterization

The textural properties of the materials were evaluated by the N_2_-adsorption isotherms at −196 °C, using an ASAP 2010 (Micromeritics, Norcross, GA, USA) apparatus (ReQuimTe Analyses Laboratory, Universidade Nova de Lisboa, Portugal). Samples were previously degassed at 300 °C for 6 h. The specific surface area (S_BET_) was calculated by the Brunauer-Emmett-Teller (BET) equation. Structural characterization was carried out by means of X-ray diffraction (XRD) in a PAN’alytical X’Pert MPD (PANanalytical, Almelo, Netherlands) equipped with a X’Celerator detector and secondary monochromator (Cu Kα, λ = 0.154 nm, 50 kV, 40 mA; data recorded at a 0.017° step size, 100 s/step), located at Universidade de Trás-os-Montes e Alto Douro, Vila Real, Portugal. The collected spectra were analyzed by Rietveld refinement using PowderCell software (by Werner Kraus and Gert Nolze, http://www.ccp14.ac.uk), allowing the determination of crystallite sizes by means of the Williamson-Hall plot. The samples were imaged by transmission electron microscopy (TEM). The analyses were performed on a Leo 906E apparatus (Austin, TX, USA), at 120 kV. Samples were prepared by ultrasonic dispersion and a 400 mesh formvar/carbon copper grid (Agar Scientific, Essex, UK) was dipped into the solution for the TEM analysis. The redox properties were assessed by temperature programmed reduction (TPR) experiments in an AMI−200 Catalyst Characterization Instrument (Altamira Instruments, Pittsburgh, PA, USA), employing H_2_ as a reducing agent. In a typical H_2_-TPR experiment, 50 mg of the sample were heated up to 1100 °C (10 °C/min), under H_2_ flow (1.5 cm^3^) balanced with He (29 cm^3^).

### 2.4. Catalytic Evaluation Studies

Catalytic tests for CO_2_ hydrogenation were carried out in a quartz fixed-bed U-shaped reactor (internal diameter, ID = 1 cm). The reactor was placed inside a high temperature furnace, equipped with a thermocouple and a Proportional Integral Differential (PID) temperature controller. In each experiment, the catalyst bed consisted of a mixture of 200 mg catalyst diluted with 200 mg of inert SiO_2_. Prior to experiments, catalysts were reduced in situ at 400 °C for 1 h under pure H_2_ flow (50 cm^3^/min), followed by flushing with He (10 cm^3^/min). The experiments were conducted at atmospheric pressure and in the temperature range of 200–500 °C at intervals of 20–25 °C. The heating rate was 1 °C/min, adequate for the establishment of steady-state conditions before each measurement. To ensure the reproducibility of the obtained conversion/selectivity values, all tests were conducted twice, without noticeable differences (<5% difference between measurements at the same temperature).

All gases used were of 99.999% purity, provided and certified by Air Liquide Hellas S.A. The total flow rate of the feed gas was 100 cm^3^/min, corresponding to a gas hourly space velocity (GHSV) of 20,000 h^−1^. Gas feed comprised of H_2_/CO_2_ mixtures at different molar ratios (1–9). The analysis of gases was performed by a gas chromatograph with He as the carrier gas, equipped with a thermal conductivity detector (TCD) for detection of CO and CO_2_, a flame ionization detector (FID) for monitoring CH_4_ and two separation columns (Molecular Sieve 13X and Porapack QS). A cold trap submerged in a water bath was connected to the reactor effluent in order to condensate H_2_O produced by the reactions.

Carbon dioxide conversion, Χ_CO_2__, and product selectivities, S_CO_ and S_CH4_, were calculated as follows (Equations (3)–(5)):

(3) $$X_{{\mathrm{CO}}_{2}}=\frac{\left({\left[{\mathrm{CO}}_{2}\right]}_{\mathrm{in}}\cdot F_{\mathrm{in}}\right)\;-\;\left({\left[{\mathrm{CO}}_{2}\right]}_{\mathrm{out}}\cdot F_{\mathrm{out}}\right)}{{\left[{\mathrm{CO}}_{2}\right]}_{\mathrm{in}}\cdot F_{\mathrm{in}}}\cdot 100$$

(4) $$S_{\mathrm{CO}}=\frac{{\left[\mathrm{CO}\right]}_{\mathrm{out}}}{{\left[\mathrm{CO}\right]}_{\mathrm{out}}+{\left[{\mathrm{CH}}_{4}\right]}_{\mathrm{out}}}\cdot 100$$

(5) $$S_{{\mathrm{CH}}_{4}}=\frac{{\left[{\mathrm{CH}}_{4}\right]}_{\mathrm{out}}}{{\left[\mathrm{CO}\right]}_{\mathrm{out}}+{\left[{\mathrm{CH}}_{4}\right]}_{\mathrm{out}}}\cdot 100$$

where [i]_in_ and [i]_out_ represent the concentrations of reactants (i = CO_2_) or products (i = CO or CH_4_) at the inlet and outlet of the reactor, respectively. F_in_ and F_out_ are the total flow rates (cm^3^/min) at the inlet and outlet of the reactor, respectively. Detection of other carbonaceous products as well as elemental carbon was either negligible or nonexistent, thus only CO_2_, CH_4_ and CO were included in the calculations of carbon balance, which closes within ±6%.

Reaction rates were defined in terms of the rate of moles of CO_2_ consumed per both mass (r_m_) and surface area (r_s_) of the catalyst:

(6) $$r_{m}\left({\mathrm{mol}\;\mathrm{CO}}_{2}\cdot g^{-1}\cdot s^{-1}\right)=\frac{{\left[{\mathrm{CO}}_{2}\right]}_{\mathrm{in}}\cdot F_{\mathrm{in}}\cdot X_{{\mathrm{CO}}_{2}}}{100\cdot 60\cdot m_{\mathrm{cat}}\cdot V_{m}}$$

(7) $$r_{s}\left({\mathrm{mol}\;\mathrm{CO}}_{2}\cdot m^{-2}\cdot s^{-1}\right)=r_{m}/S_{\mathrm{BET}}$$

where m_cat_ (g) is the mass of the catalyst, S_BET_ (m^2^/g) is the surface area of the catalyst and V_m_ is the gas molar volume at 25 °C and 1 bar (24,436 cm^3^/mol).

The thermodynamic equilibrium calculations were elaborated using the minimization of Gibbs free energy mathematical model (RGibbs) in Aspen Plus^®^ software (Aspen Technology, Inc., Bedford, MA, USA) and the simulation results are included in the catalytic performance plots. The following components were included in the model: CO_2_, H_2_, CO, H_2_O, CH_4_, CH_3_OH, CH_3_OCH_3_, HCOOH, C_2_H_4_ and C_2_H_6_. Only the first five substances appeared to be formed in a significant amount in the equilibrium mixture, when all possible reactions between CO_2_ and H_2_ were considered. Properties of the substances were determined using the Soave–Redlich–Kwong (SRK) equation of state, a widely used method in gas-processing simulation.

## 3. Results and Discussion

### 3.1. Morphological Characterization (TEM)

**Figure 1:**
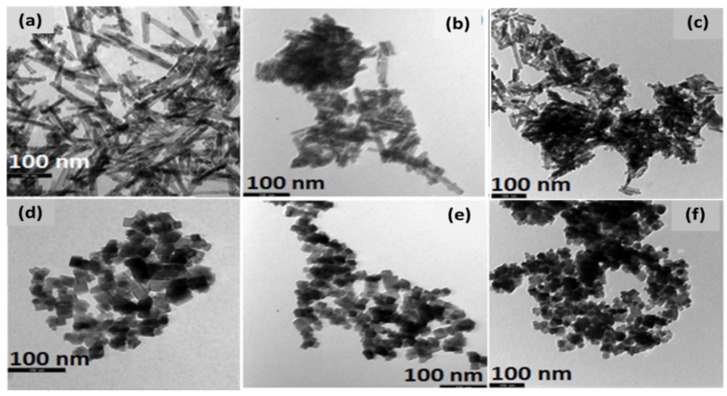
Transmission electron microscope (TEM) images of the samples: (**a**) CeO_2_-NR, (**b**) Cu/CeO_2_-NR, (**c**) Co/CeO_2_-NR, (**d**) CeO_2_-NC, (**e**) Cu/CeO_2_-NC, (**f**) Co/CeO_2_-NC.

In order to gain insight into the morphology of bare CeO_2_ and M/CeO_2_ samples, TEM analysis was performed. The NR samples (Figure 1a–c) display ceria in a uniform rod-like morphology, while the existence of nanocubic morphology is evident in the samples denoted as NC (Figure 1d–f). Apparently, the incorporation of the active metal phase into the CeO_2_ lattice has no effect on the support morphology, since distinctive rod and cubic particles are still clearly observed after the incorporation of the metal phase on the well-defined support structure. This also confirms the findings of XRD studies, where the structural features of catalysts remained essentially unaffected after the incorporation of cobalt and copper into the ceria support.

### 3.2. Textural and Structural Characterization (Brunauer–Emmett–Teller (BET), X-ray Diffraction (XRD))

The textural, structural and redox properties of bare ceria as well as of M/CeO_2_ catalysts are presented in Table 1. Bare ceria supports, i.e., CeO_2_-NR and CeO_2_-NC, exhibit a BET surface area of 79 and 37 m^2^/g, respectively. The incorporation of transition metals into ceria carriers slightly decreases the BET area. However, the order obtained for bare supports remained unaffected, that is, nanorod samples exhibit higher surface area than the corresponding nanocubic ones, regardless of the nature of the metal phase incorporated into the ceria carrier.

**Figure 2:**
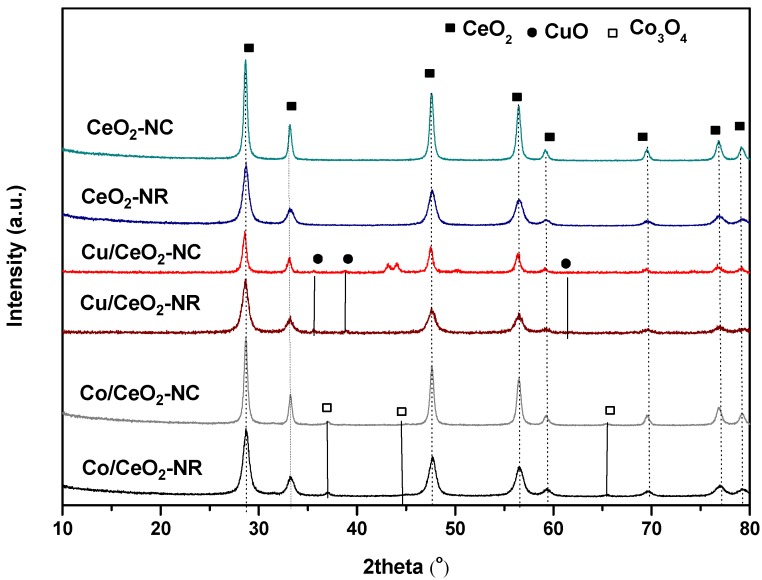
X-ray diffraction (XRD) patterns of the as-prepared samples.

According to XRD results (Figure 2), the main peaks for all samples at 28.5°, 33.1°, 47.5°, 56.3°, 59.1°, 69.4°, 76.7°, and 79.1° correspond to the (111), (200), (220), (311), (222), (400), (331) and (420) planes of a face-centered cubic (FCC) fluorite structure of ceria (Fm3m symmetry, no. 225) [69] with (111), (220) and (311) planes mostly prevailing in the samples structure. Moreover, all M/CeO_2_ samples exhibit smaller peaks indexed to the corresponding oxide due to the incorporation of metal phase (~8 wt.% Co or Cu) into ceria support. Peaks corresponding to CuO crystal phases at 2θ = 35.3°, 38.2° and a less distinguishable peak at 62° are observed for Cu/CeO_2_ samples, indicating heterodispersion or aggregation of copper species on the surface of ceria [70]. For Cu/CeO_2_-NC, the double peak at 43–44° as well as the peak at 50° are present due to the sample holder. The Co/CeO_2_ samples show small peaks characteristic of Co_3_O_4_ at 2θ ~ 36°, 44° and 66° [71].

The average crystallite size of CeO_2_, CuO and Co_3_O_4_ phases for both bare ceria and M/Ceria samples of different morphology are summarized in Table 1. Ceria particles are following the order: CeO_2_-NC (27 nm) > Co/CeO_2_-NC (24 nm) > Cu/CeO_2_-NC (19 nm) > CeO_2_-NR (15 nm) > Co/CeO_2_-NR (14 nm) > Cu/CeO_2_-NR (12 nm). These values are close to those reported in literature for ceria-supported materials [52,72]. It is worth mentioning that ceria crystallite size of all samples with nanocubic morphology is higher than that of the corresponding samples with rod-like morphology. Moreover, no significant changes were observed on ceria particle sizes upon the incorporation of Cu or Co, implying that the structural features of ceria remain unaffected by metal addition, as further verified by TEM analysis (see above). Regarding the crystallite size of active metal phases, the CuO size is 52 and 43 nm for Cu/CeO_2_-NC and Cu/CeO_2_-NR, respectively. The corresponding values for Co_3_O_4_ phase are much lower, i.e., 19 and 16 nm, denoting a better dispersion of cobalt compared to copper on ceria support. Similar conclusions have been previously obtained in a series of CeO_2_ supported transition metal catalysts (Fe, Co, Ni, Cu) synthesized by the incipient wet impregnation method [73]. It is also worth mentioning that the crystallite size of both active phases (Co_3_O_4_ or CuO) and ceria nanoparticles follow the same trend, implying that the structural characteristics of CeO_2_ can determine the particle size of metal oxides (Co_3_O_4_ or CuO), in agreement with relevant literature studies [74,75]. Moreover, it should be noted that the crystallite size of both ceria and Co_3_O_4_ or CuO phases follows the reverse order of BET area, indicating an agglomeration upon the decrease of surface area.

### 3.3. Redox Properties (Hydrogen Temperature-Programmed Reduction (H_2_-TPR))

**Figure 3:**
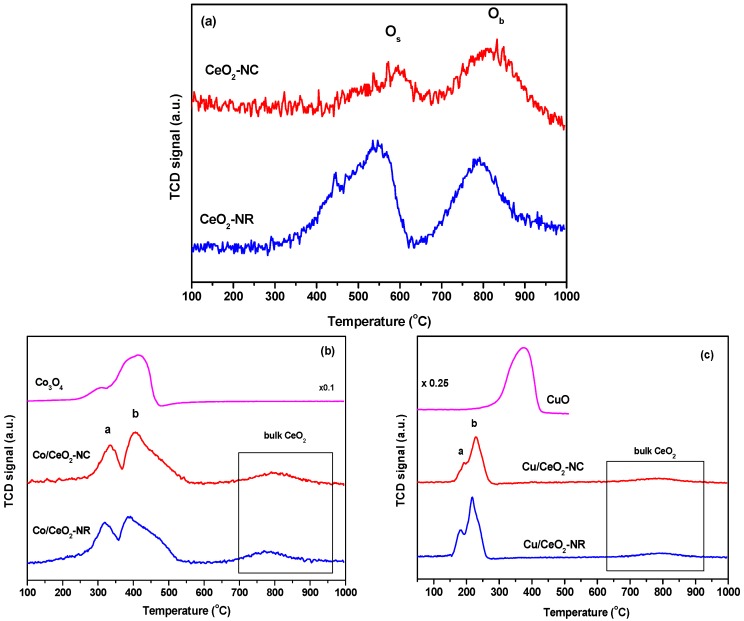
Hydrogen temperature-programmed reduction (H_2_-TPR) profiles of the as-prepared samples; (**a**) CeO_2_-NX, (**b**) Co/CeO_2_-NX, (**c**) Cu/CeO_2_-NX. H_2_-TPR profiles of bare Co_3_O_4_ (**b**) and CuO (**c**) oxides are also depicted for comparison purposes.

The redox properties of as-prepared catalysts were evaluated by means of hydrogen temperature-programmed reduction (H_2_-TPR) studies. Figure 3 presents the reduction profiles of all samples. In Table 1 the redox properties, in terms of H_2_ consumption (mmol/g) and maximum temperature of main TPR peaks, are summarized. The reduction profiles of bare CeO_2_ samples (Figure 3a) consist of two broad peaks centered at 545–590 °C and 790–810 °C, which are attributed to ceria surface (O_s_) and bulk oxygen (O_b_) reduction, respectively [76,77]. Both peaks are exhibited in lower temperatures for the nanorod CeO_2_ with the effect being more intense for the ceria surface oxygen. Similar behavior is displayed also for the impregnated Co- and Cu-based samples, where both peaks are shifted to substantially lower temperatures, indicating the beneficial role of active phases in the overall redox properties of the as-prepared samples.

The corresponding TPR profiles of Co/CeO_2_ and Cu/CeO_2_ samples along with those of bare Co_3_O_4_ and CuO, are shown in Figure 3b,c, respectively. Interestingly, all M/CeO_2_ samples exhibit reduction peaks at significantly lower temperatures than bare oxides, which implies the synergy between the metal phase and CeO_2_ towards an improved reducibility. In particular, the Co/CeO_2_ samples exhibit two main reduction peaks at 318–335 °C (peak a) and 388–405 °C (peak b), attributed to the subsequent reduction of Co_3_O_4_ to CoO and CoO to metallic Co, respectively [78]. As for the Cu/CeO_2_ samples, the low-temperature peak (peak a) in the range of 181–194 °C is attributed to the reduction of finely dispersed CuO_x_ species strongly interacting with the ceria surface [68]. The peak at higher temperature (peak b) can be attributed to the formation of larger CuO clusters on the ceria surface [79].

It is obvious that the reduction of M/CeO_2_ samples occurs at a significantly lower temperature than those of bare ceria samples, due to the synergy between the two oxide phases that weakens the metal-oxygen bonds [80]. It is also of worth pointing out the facilitation of the ceria surface oxygen reduction in the presence of metal phase (Co or Cu), which results in overlapping bands in the low-temperature range of the TPR profiles of M/CeO_2_ samples (Figure 3b,c).

In order to gain further insight into the impact of support morphology, as well as of the nature of the active phase on the redox properties of the as-prepared samples, the H_2_ consumption in the low temperature range (100–600 °C), corresponding to the surface oxygen reduction of both active phase and ceria, has been estimated (Table 1). CeO_2_-NR exhibits higher values (0.59 mmol H_2_·g^−1^) than CeO_2_-NC (0.41 mmol H_2_·g^−1^), implying the enhanced reducibility and oxygen mobility of ceria-nanorods. These findings are in complete agreement with the in situ Raman analysis performed in our previous work, in which nanorods exhibited the highest amount in defects and oxygen vacancies [68]. In a similar manner, the M/CeO_2_ samples of nanorod morphology exhibit higher H_2_ consumption than nanocubes in all cases, indicating their abundance in loosely-bound oxygen species. Regarding the impact of metal entity, the total hydrogen consumption follows, independently on support morphology, the order: Co/CeO_2_ > Cu/CeO_2_ > CeO_2_, matching the catalytic performance in terms of CO_2_ conversion (see below). It should also be noted that hydrogen consumption exceeds the theoretical amount required for the complete reduction of the oxidized transition metal phases for all M/CeO_2_ samples (Table 1). The latter implies the facilitation of ceria capping oxygen reduction in the presence of a metal entity, further corroborating the synergistic effect between the metal and ceria carrier.

### 3.4. Catalytic Evaluation Studies

#### 3.4.1. CO_2_ Hydrogenation Activity

**Figure 4:**
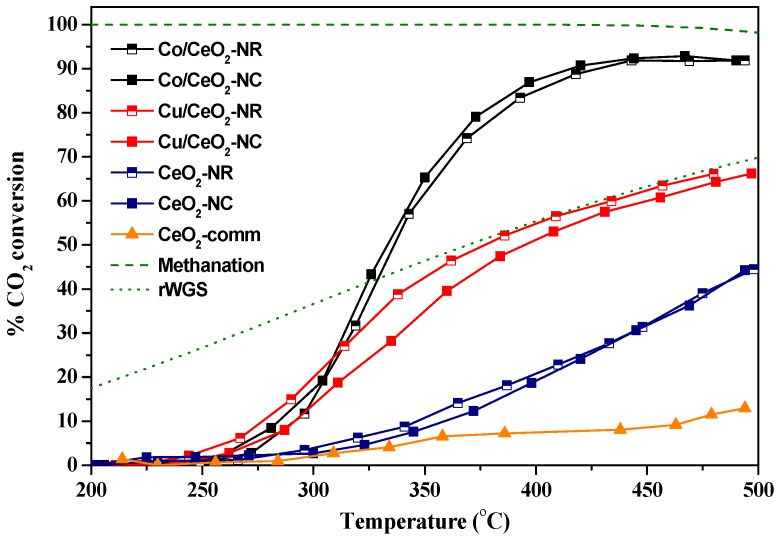
Experimental (solid lines) and theoretical (green lines) CO_2_ conversion profiles for commercial CeO_2_, bare ceria-NX and M/CeO_2_-NX samples in CO_2_ hydrogenation reaction. The dotted and dashed green line correspond to reverse water-gas shift (rWGS) and methanation reactions equilibrium, respectively. (F = 100 cm^3^/min, gas hourly space velocity (GHSV) = 20,000 h^−1^, H_2_:CO_2_ = 9:1, P = 1 atm).

The catalytic performance of the as-prepared samples in the CO_2_ hydrogenation reaction was investigated in the temperature range of 200–500 °C. A commercial CeO_2_ sample (Fluka, S_BET_ = 15 m^2^/g), denoted as CeO_2_-comm, was also tested as a reference sample. Figure 4 depicts CO_2_ conversion of all samples in the temperature range investigated, as compared to the thermodynamic equilibrium CO_2_ conversion profiles for methanation and rWGS reactions. In Table 2 the results from catalytic evaluation studies, in terms of both CO_2_ conversion and normalized rates at 400 °C, are presented comparatively for all samples.

The beneficial effect of the synthesis method on the catalytic activity is obvious, as all nano-ceria samples exhibit far better CO_2_ conversion values than CeO_2_-comm. Moreover, the incorporation of a metal phase into ceria leads to a dramatic improvement of the catalytic activity. Specifically, Co/CeO_2_-NX, Cu/CeO_2_-NX, CeO_2_-NX and CeO_2_-comm catalysts exhibited CO_2_ conversion values at 400 °C of approximately 86%, 52%, 20% and 8%, respectively. The observed order of CO_2_ conversion generally correlates with the amount of consumed hydrogen calculated by H_2_-TPR in Table 1.

The increase in temperature obviously increases the CO_2_ conversion, but to a different extent for each sample. As expected, CO_2_ conversion initially increases and reaches a plateau for the highly selective towards methane Co/CeO_2_-NX catalysts, favoring the exothermic CO_2_ methanation (Equation (2)) below 500 °C, in agreement with the thermodynamic calculations. A different trend was shown for bare ceria and Cu/CeO_2_-NX samples, as CO_2_ conversion increases steadily, but to a lesser extent, as compared to Co/CeO_2_-NX samples, approaching in the case of Cu-based catalysts the equilibrium for the rWGS reaction. Bare ceria carriers, although clearly favoring the rWGS reaction, demonstrated CO_2_ conversion values that are well below the corresponding values predicted by thermodynamics.

**Figure 5:**
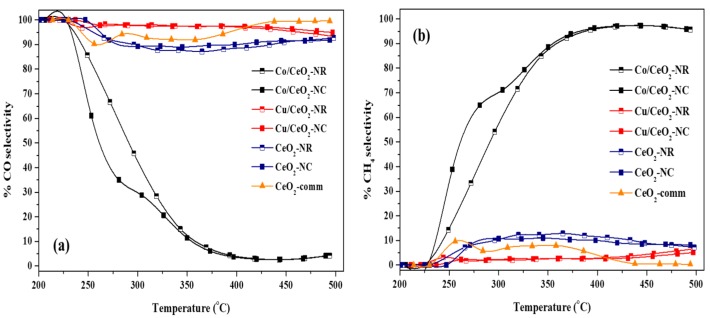
Selectivity to (**a**) CO and (**b**) CH_4_ as a function of temperature for all samples in the CO_2_ hydrogenation reaction. (F = 100 cm^3^/min, GHSV = 20,000 h^−1^, H_2_:CO_2_ = 9:1, P = 1 atm).

The impact of the nature of the metal phase (Cu, Co) on the CO_2_ hydrogenation performance is further evaluated on the basis of the selectivity towards CH_4_ and CO, depicted in Figure 5. Apparently, commercial ceria, nano-ceria and Cu/CeO_2_-NX samples are all highly selective to CO (>90%). Thus, maximum CO_2_ conversion values for these samples are expected to be closer to those for the rWGS reaction equilibrium, a much less favorable reaction than CO_2_ methanation, as indeed shown by the corresponding equilibrium curve (dotted line) in Figure 4. Similar results for CO production over CeO_2_ [59,81] and Cu-based catalysts are reported in literature (see Table 3). Interestingly, Cu/CeO_2_-NR reaches equilibrium conversion values at ca. 380 °C, well below that reported for many rWGS catalysts. On the contrary, the addition of cobalt into CeO_2_ leads to a completely different trend, since the selectivity towards CH_4_ for all Co/CeO_2_ samples is approximately 95% at temperatures higher than ca. 400 °C. However, at temperatures below 400 °C, selectivity towards CO is significant, decreasing rapidly for higher temperatures.

In light of the above results, it should be highlighted that the as-prepared cobalt catalysts are highly efficient in methane production at temperatures as low as 400 °C, under the reaction conditions employed, being superior, or at least comparable, to the most active Co-based catalysts reported in literature (see Table 3). On the other hand, the as-prepared copper catalysts are highly selective to CO, reaching rWGS equilibrium values at ca. 380°C. In a similar manner, Dai et al. [82] reported 100% selectivity to CO for Cu/CeO_2_, attributed to the presence of Cu^0^ active sites, whereas selectivity to CH_4_ for Co/CeO_2_ increased markedly between 350°C and 400 °C.

The obtained differences in activity/selectivity between Cu- and Co-based catalysts can be corroborated by taking into account the underlying mechanism of Cu- and Co-catalyzed CO_2_ hydrogenation reaction, in conjunction with the present characterization results. In particular, the dissociation of adsorbed CO, considered as the rate-determining step of the CO_2_ methanation process [92,93], has been proposed to proceed by two main pathways: direct CO_ads_ dissociation and H-assisted CO_ads_ dissociation. The first mechanism is proposed to occur over group VIII metal-based catalysts, such as Co [94,95]. In view of this fact, Liu et al. [48] demonstrated that Co showed more favorable thermodynamics and lower CO_2_ decomposition barriers for CO_2_ reduction compared to Cu. In a similar manner, a close relationship between the CO_2_ and H_2_ adsorption capacity of Co/KIT-6 catalysts and their CO_2_ conversion/selectivity performance has been revealed [87]. In particular, high H_2_ adsorption capacity can provide a large number of active H species for the further hydrogenation of intermediate species (such as HCOO^−^) to methane, whereas the low H_2_ adsorption and activation capacity is favorable to CO formation. Considering the fact that as-prepared Co/CeO_2_-NX possess a higher reducibility than Cu/CeO_2_-NX in terms of H_2_ uptake (Table 1), this could be an explanation for the high methane selectivity exhibited for the Co-based samples.

By contrast, a redox mechanism has been proposed in the literature for rWGS reaction over Cu-based catalysts [29,96]. Specifically, Cu^0^ atoms can act as active sites for the dissociation of CO_2_ and the Cu_2_O formed is subsequently reduced by hydrogen to regenerate metallic Cu species. Hydrogen was proposed to be only a reducing agent without direct participation in the formation of intermediate species in the rWGS reaction [97]. The scheme can be simplified to the following reactions (Equations (8) and (9)):

CO_2_ + 2Cu → Cu_2_O + CO (8)

H_2_ + Cu_2_O → 2Cu + H_2_O (9)

The facile reduction of Cu/CeO_2_-NX catalysts towards reduced copper species at temperatures lower than ca. 300 °C, corroborated by the H_2_-TPR results (Figure 3), can possibly favor this redox mechanism, thus being the reason for the high selectivity towards CO even at low temperatures. However, hydrogenation of carbon dioxide proceeds through a rather complex pathway, thus more detailed work has to be undertaken in order to elucidate the differences in the mechanisms of both CO_2_ methanation and rWGS and in the product distribution depending on the metal active phase of the catalyst. To this end, mechanistic studies are currently under development in our laboratory.

Regarding the impact of support morphology, it can be concluded that the CO_2_ conversion and selectivity of the samples are not significantly affected by the support morphology (NR vs. NC), as shown in Figure 4 and Figure 5. In particular, a slightly better conversion performance is obtained for Cu/CeO_2_-NR samples as compared to Cu/CeO_2_-NC, whereas for bare CeO_2_ and Co/CeO_2_ minor differences in conversion performance between NC and NR were observed. This can also be demonstrated by the mass-normalized reactions rates, as values for NRs are very close to those for NCs (Table 2).

It should be mentioned, however, that NC samples exhibit two to three times lower BET surface area as compared to NR samples (Table 1), which should be further accounted for the different catalytic activity of NC vs. NR samples. In order to more precisely gain insight into the impact of support morphology on the intrinsic reactivity, the specific activity normalized per unit of surface area (µmol CO_2_·m^−2^·s^−1^) was thus calculated (Equation (7)) and is summarized in Table 2. It is evident that the area-normalized reaction rates of nanocubic samples are considerably higher compared to their nanorod counterparts. Specifically, Co/CeO_2_-NC exhibits the highest rate (1.07 µmol CO_2_·m^−2^·s^−1^), followed by Cu/CeO_2_-NC (0.51 µmol CO_2_·m^−2^·s^−1^) and CeO_2_-NC (0.18 µmol CO_2_·m^−2^·s^−1^). Samples with nanorod morphology exhibit almost half of the reaction rates shown by the nanocubic samples, with values of 0.40, 0.25 and 0.09 µmol CO_2_·m^−2^·s^−1^ for Co/CeO_2_-NR, Cu/CeO_2_-NR and CeO_2_-NR, respectively.

In a similar manner, it was found that Ru catalysts supported on ceria nanocubes showed higher CO_2_ methanation activity, in comparison with ceria nanorods and nanopolyhedra, on the basis of specific rate [98]. Kovacevic et al. [81] synthesized CeO_2_ nanoparticles with distinct morphology for the rWGS reaction and reported that nanocubes were more active than nanorods in terms of CO produced per surface area, owing to the greater inherent reactivity of (100) crystal planes enclosing cubes, in contrast with the inherently less reactive (111) facets exposed by rods and particles.

#### 3.4.2. Effect of H_2_:CO_2_ Ratio

The effect of the H_2_:CO_2_ feed ratio (9:1, 6:1, 4:1 and 1:1) on the conversion and selectivity performance was next explored over Co/CeO_2_-NR and Cu/CeO_2_-NR samples. Reactant feed ratio is an important factor in the CO_2_ hydrogenation process, as it modifies the thermodynamic equilibrium of the system [99] and could increase selectivity of a specific product [10,100]. Moreover, knowledge on a catalyst performance under different reaction mixtures is important, especially with the scope of employing the catalytic system in an industrial-scale process, where hydrogen flow may vary, due to the inherent fluctuations associated with the available excess RES power [101].

**Figure 6:**
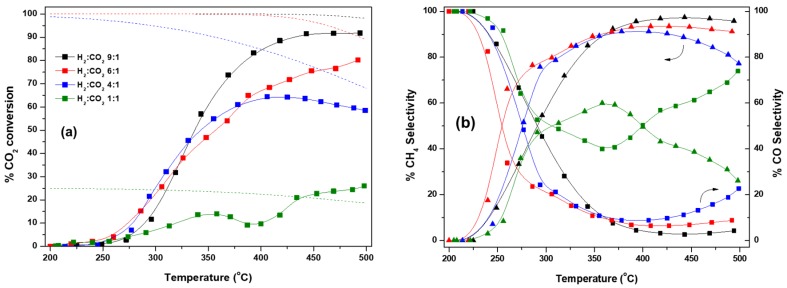

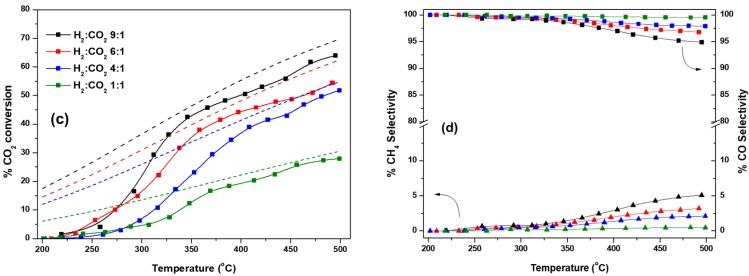
Effect of H_2_:CO_2_ ratio on theoretical and experimental CO_2_ conversion and selectivity to CH_4_ (triangle) and CO (square) values for (**a**,**b**) Co/CeO_2_-NR and for (**c**,**d**) Cu/CeO_2_-NR. Dotted and dashed lines represent equilibrium CO_2_ conversion values for the methanation and rWGS reactions, respectively.

The CO_2_ conversion along with the selectivities to CH_4_ and CO, as a function of temperature, for the different H_2_/CO_2_ feed molar ratios investigated, are depicted in Figure 6. The corresponding equilibrium curves for each reaction system are also shown for comparison. It is evident that the increase of H_2_:CO_2_ ratio exerted a beneficial effect on the CO_2_ conversion values, according to thermodynamics.

In particular, for Co/CeO_2_-NR catalysts, CO_2_ conversion values at 450 °C of 23, 61, 81, and 90% at a H_2_:CO_2_ ratio of 1, 4, 6 and 9, were obtained, respectively. When equal amounts of CO_2_ and H_2_ were fed into the reactor, CO_2_ conversion first increased up to 14% at 360 °C, decreased until 400 °C and then re-increased to a value of 26% at 500 °C. This behavior is expected thermodynamically, as sub-stoichiometric conditions for CO_2_ methanation were employed and rWGS was thus favored, allowing the conversion to exceed the theoretical quasi-limit calculated by including only CH_4_ as a product (green dotted line in Figure 6a). Similar results were found on thermodynamic analyses for CO_2_ methanation in literature [36,100,102]. It is worth mentioning that Co/CeO_2_-NR almost reaches maximum CO_2_ conversion values at ca. 400 °C for over-stoichiometric conditions and ca. 420 °C in the case of stoichiometric ratio. In addition, CO_2_ conversion approached values as high as 90% of the thermodynamic equilibrium ones, implying the superiority of Co/CeO_2_ catalysts for the Sabatier reaction.

Higher H_2_:CO_2_ feed ratios are likely to promote methane formation instead of the reverse WGS reaction, since Equation (2) is more dependent on hydrogen. Indeed, at 450 °C, selectivity values towards CH_4_ equal to 97.5%, 93.6%, 88.2%, and 38.1% were observed for a feed ratio of 9:1, 6:1, 4:1 and 1:1, respectively (Figure 6b). CH_4_ selectivity decreased significantly at temperatures higher than 380 °C when the H_2_:CO_2_ ratio was unity, reaching a value of 26% at 500 °C. The opposite trend was observed for CO selectivity. Traces of C_2_ hydrocarbons were also observed for Co/CeO_2_-NR, especially under H_2_-limiting conditions. In general, significant CO_2_ conversion to CH_4_ can be achieved on Co/CeO_2_-NR, even when hydrogen availability decreases, as long as the system is operating far from sub-stoichiometry.

For Cu/CeO_2_-NR, CO_2_ conversion at 450 °C increased from 24.2% to 58.1% as the feed ratio increased from 1:1 to 9:1, respectively, in agreement with the equilibrium CO_2_ conversion to CO. Considering that equimolar amounts of CO_2_ and H_2_ are required for the stoichiometric rWGS reaction to take place, any excess amount of hydrogen in the feed is expected to facilitate CO production. Regardless of the hydrogen inlet concentration, CO_2_ conversion approached the respective equilibrium values within ~5% at around 380 °C. Several catalytic systems employed for rWGS have been reported to function under thermodynamic limitation at the medium-high or high temperature regime [103,104,105]. As for the selectivity to CH_4_, it increased slightly, from about 1.0% for a H_2_:CO_2_ ratio of 1:1 to 5.1% for a ratio of 9:1. Correspondingly, CO selectivity decreased slightly with an increase in H_2_:CO_2_ ratio, with selectivity values at 500 °C equal to 95.3%, 97.1%, 98.2% and 99.5%, at H_2_:CO_2_ ratios of 9, 6, 4 and 1, respectively. Contrary to Co/CeO_2_-NR, no other carbonaceous products were identified in the case of Cu/CeO_2_, at the reaction conditions examined. Low methane selectivity values exhibited by Cu/CeO_2_-NR, even under excess hydrogen in the reactant stream, further substantiate the possibility of a redox mechanism over this catalyst. Excess hydrogen may directly re-reduce Cu_2_O species formed by CO_2_ dissociation over metallic Cu, rather than fully hydrogenating intermediate species, thus leading to high CO production, as discussed above.

#### 3.4.3. Stability Tests

**Figure 7:**
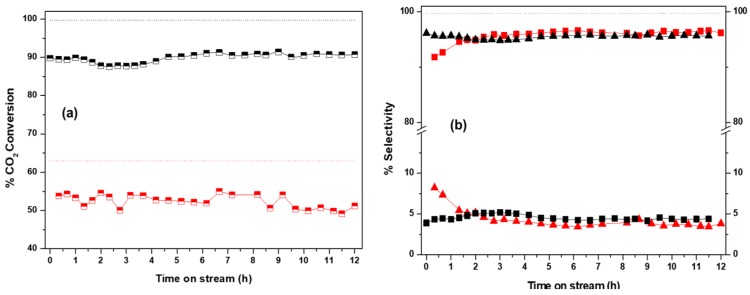
Dependence of (**a**) CO_2_ conversion and (**b**) selectivity to CO (square) and CH_4_ (triangle) on time on stream for Cu/CeO_2_-NR (red) and Co/CeO_2_-NR (black). Dotted lines refer to the corresponding equilibrium conversions. Experimental conditions: T = 450 °C, H_2_:CO_2_ = 9:1, P = 1 atm, GHSV = 20,000 h^−1^.

Further examination of the catalytic performance of Co/CeO_2_-NR and Cu/CeO_2_-NR was conducted in short-term stability experiments under isothermal conditions, in order to assess the catalysts lifetime characteristics and to detect possible deactivation phenomena. Herein, the aforementioned samples were further tested for 12 h at a constant temperature of 450 °C, with a feed ratio of H_2_:CO_2_ equal to 9:1 and a GHSV of 20,000 h^−1^. The results of CO_2_ conversion and selectivity values as a function of time are presented in Figure 7.

Copper-based catalysts are generally known for their inferior catalytic performance under high-temperature operation, due to the sintering of copper particles resulting in a decrease in active sites [106,107]. However, CO_2_ conversion on Cu/CeO_2_-NR remained relatively stable for the duration of the experiment, fluctuating between an average value of 52%, decreasing very slightly from the value of 56% found for the fresh catalyst during the corresponding light-off tests. It is also noted that the thermodynamic value for the rWGS reaction, under the employed conditions, is 63%. Moreover, the selectivity towards CO stabilized to ca. 96% after approximately 1 h. Thus, the Cu/CeO_2_-NR remained highly active towards CO production and exhibited very low methane yield even after 12 h at 450 °C, under hydrogen excess conditions, further corroborating the fact that CO_2_ methanation is largely unfavorable over this catalyst.

In the course of the methanation reaction, the stability of a catalyst is closely linked to coking and metal sintering [108,109]. For Co/CeO_2_-NR, it was demonstrated that CO_2_ conversion remained very stable at ~90%, whereas the thermodynamic value is calculated at 99.5% (Figure 7). At the same time, the selectivity towards CH_4_ remained stable at 96% for the whole duration of experiment, revealing the superiority of Co/CeO_2_-NR sample in CO_2_ methanation process, in terms of conversion, selectivity and stability.

## 4. Conclusions

The present results revealed the strong effect of metal phase (Cu or Cu) and reaction conditions on the CO_2_ hydrogenation performance of nanoceria-based M/CeO_2_ catalysts of different morphology (nanorods or nanocubes). It was shown that hydrothermally synthesized nano-ceria carriers exhibited better catalytic activity in the hydrogenation of CO_2_ than commercial ceria. More importantly, incorporating Co and Cu into the nanostructured ceria support led to a significant increase in the catalytic activity, with CO_2_ conversion following the order Co/CeO_2_ > Cu/CeO_2_ > CeO_2_. This order correlates with the hydrogen consumption estimated by H_2_-TPR, implying that the enhanced reducibility—linked to the different metal phase—probably favors the CO_2_ hydrogenation reactivity. The main product of the reaction was determined by the nature of the metal entity incorporated into cerium oxide, with Co/CeO_2_ exhibiting high selectivity towards CH_4_ and CO_2_ conversion values close to equilibrium. Cu/CeO_2_ exhibited remarkable CO selectivity even under hydrogen excess conditions and very close to equilibrium CO_2_ conversion values for the rWGS reaction. From a practical point of view, Co/CeO_2_ catalysts demonstrated an excellent conversion and selectivity performance, offering ~85% yield to methane at 450 °C. On the other hand, Cu/CeO_2_ samples were very selective for CO production, exhibiting 52% CO_2_ conversion and 95% CO selectivity at ca. 400 °C.

In both cases a stable conversion and selectivity performance (either for CO_2_ methanation or rWGS reaction) was attained in short-term (12 h) stability tests. Regarding the impact of ceria morphology, the samples supported on ceria nanocubes exhibited higher specific activity (µmol CO_2_·m^−2^·s^−1^), as compared to samples of rod-like shape, revealing the significant role of support morphology, besides that of metal nature (Co or Cu). These results are considered to be very promising in the sense of employing these catalysts in real-scale processes with variable H_2_:CO_2_ ratios, where renewable hydrogen from excess RES power can be used to efficiently and selectively convert CO_2_ to CO or CH_4_.

## Figures and Tables

**Table 1:** Textural, structural and redox properties of the as-prepared samples.

Sample	BET Analysis	XRD Analysis		H_2_-TPR Analysis			
	S_BET_ (m^2^/g)	Average Crystallite Diameter, D_XRD_ (nm)		H_2_ Consumption (mmol H_2_ g^−1^) ^1^	Theoretical H_2_ (mmol H_2_ g^−1^) ^2^	Peak Temperature (°C)	
		CeO_2_	Co_3_O_4_/CuO				
CeO_2_-NC	37	27	-	0.41	-	589	809
CeO_2_-NR	79	15	-	0.59	-	545	788
Co/CeO_2_-NC	28	24	19	2.05	1.76	335	405
Co/CeO_2_-NR	72	14	16	2.37	1.76	318	388
Cu/CeO_2_-NC	34	19	52	1.50	1.34	194	228
Cu/CeO_2_-NR	75	12	43	1.80	1.34	181	217

^1^ Estimated by the quantification of H_2_ uptake in the low temperature range (100–600 °C) of the TPR profiles. ^2^ Calculated as the amount of H_2_ required for the complete reduction of Co_3_O_4_ to Co and CuO to Cu.

**Table 2:** Conversion (X_CO2_), selectivity (S) and specific activity (r_s_, r_m_) of investigated samples at 400 °C.

Sample	% X_CO2_	% S_CO_	% S_CH4_	Reaction Rates	
				r_s_ (µmol CO_2_·m^−2^·s^−1^)	r_m_ (µmol CO_2_·g^−1^·s^−1^)
CeO_2_-NR	21.1	88.5	11.5	0.09	7.2
CeO_2_-NC	19.3	89.8	10.2	0.18	6.6
Cu/CeO_2_-NR	55.0	97.0	3.0	0.25	18.8
Cu/CeO_2_-NC	50.1	97.5	2.5	0.51	17.1
Co/CeO_2_-NR	84.9	5.5	94.5	0.40	28.9
Co/CeO_2_-NC	87.7	3.7	96.3	1.07	29.9

**Table 3:** Comparison of the CO_2_ reduction performance of Cu- and Co-based catalysts for the rWGS and CO_2_ methanation reactions, respectively, at atmospheric pressure.

Sample	T (°C)	% X_CO2_	% S_CO_	% S_CH4_	H_2_:CO_2_	%wt. Cu or Co	Ref.
Cu-Catalyzed rWGS Reaction							
Cu/CeO_2_-NR	400	19	99.6		1	8.5	This work
		38	99.0		4		
Fe-Cu/Al_2_O_3_	400	36	89		4	8.2	[15]
Cu/CeO_2_	400	31.3	100		4	13	[82]
Cu/CeO_2_-NR	450	49	N/A		5	5	[83]
Cu/CeO_2_	300	~18	100		3	9	[60]
Cu-Ni/γ-Al_2_O_3_	500	23.2	75.5		1	15	[84]
Cu-Fe/SiO_2_	600	15	N/A		1	10	[85]
Cu/β-Mo_2_C	400	16	97.6		2	1.3	[86]
Co-Catalyzed CO_2_ Methanation							
Co/CeO_2_-NR	400	62.8		91.1	4	7.9	This work
		84.9		94.5	9		
Co/CeO_2_	400	34.9		37	4	10	[82]
Co/SiO_2_	360	44.3		86.5	4	10	[87]
Co/KIT-6	300	51		98.9	4.6	20	[88]
Ni-Co/Ce_0.25_Zr_0.75_O_2_	280	85		98	4	5	[89]
Co/Al_2_O_3_	300	38		100	4	15	[90]
Co/CeO_2_	300	97		~96	9	42.3	[91]
